# Dataset of ligand-controlled synthesis of CsPbBr_3_ nanoplatelets

**DOI:** 10.1016/j.dib.2022.107997

**Published:** 2022-02-25

**Authors:** Junsang Cho

**Affiliations:** Department of Chemistry, Duksung Women's University, Seoul 01369, South Korea

**Keywords:** Lead halide perovskites, Perovskite nanocrystals, CsPbBr_3_ nanoplatelets, Ligand-mediated synthesis

## Abstract

Lead halide perovskites nanocrystals have emerged as next-generation materials in the application of photovoltaics and various optoelectronics owing to the controllable optoelectronic properties (achieved through varying the dimensionality and composition of the materials). The design and control to obtain the desirable optoelectronic properties of the halide perovskite nanocrystals, thus, remain paramount importance. The synthesis and stabilization of cesium lead bromide (CsPbBr_3_) nanoplatelets through ligand-assisted reprecipitation protocol (LARP) can indeed enable the manipulation of the layer thickness over the resulting nanoplatelets. Herein, we have elucidated the role of ligand concentration and chain length effect on the crystal growth and mapped how these parameters affect the layer thickness (and crystal growth kinetics) of the corresponding nanoplatelets. Complex mapping the evolution of the average layer thickness of the CsPbBr_3_ nanoplatelets provide a detailed perspective of the crystal growth with ligand shell assembly. Transmission electron microscopy (TEM) images directly measurable for the thickness of nanoplatelets along with photoluminescence (PL) emission spectroscopy have been employed to determinate of the thickness of the nanoplatelets exhibiting thickness-dependent optical properties with different layer thickness nanoplatelets.

## Specifications Table

**Table:** 

Subject	Materials Science and Engineering / Inorganic chemistry
Specific subject area	Lead halide perovskite nanoplatelets
Type of data	Scheme Image Graph
How data were acquired	Transmission electron microscope, Photoluminescence emission spectroscopy
Data format	Raw Analysed
Parameters for data collection	Colloidal solution of cesium lead halide perovskite nanocrystal dispersed in toluene were recorded for photoluminescence emission spectra
Description of data collection	Photoluminescence emission spectra were recorded in the spectral range of 400-600 nm under the excitation wavelength of 360 nm using a Xenon arc lamp as the light excitation source
Data source location	Institution: Duksung Women's University City/Town/Region: Seoul Country: South Korea Latitude and longitude (and GPS coordinates, if possible) for collected samples/data:
Data accessibility	https://data.mendeley.com/datasets/7hn8v6czkf/1
Related research article	***For a published article:*** *J. Cho, S. Banerjee, Ligand-Directed Stabilization of Ternary Phases: Synthetic Control of Structural Dimensionality in Solution-Grown Cesium Lead Bromide Nanocrystals, Chem Mater. 30 (2018) 6144–6155.*https://doi.org/10.1021/acs.chemmater.8b02730

## Value of the Data

• The data shown herein explains the structural evolution for cesium lead halide perovskites as a function of varying the ligand concentration and ligand chain length.
• The data provides a fundamental insight on the layer thickness-control that is mediated through a surface-passivating ligand for stabilizing the cesium lead halide nanoplatelets.
• The data can be further used for designing experiments and advanced research in the field of synthesis of halide perovskite nanoplatelets and their application in various optoelectronic devices.

## 1 Data Description

**Fig. 1:**
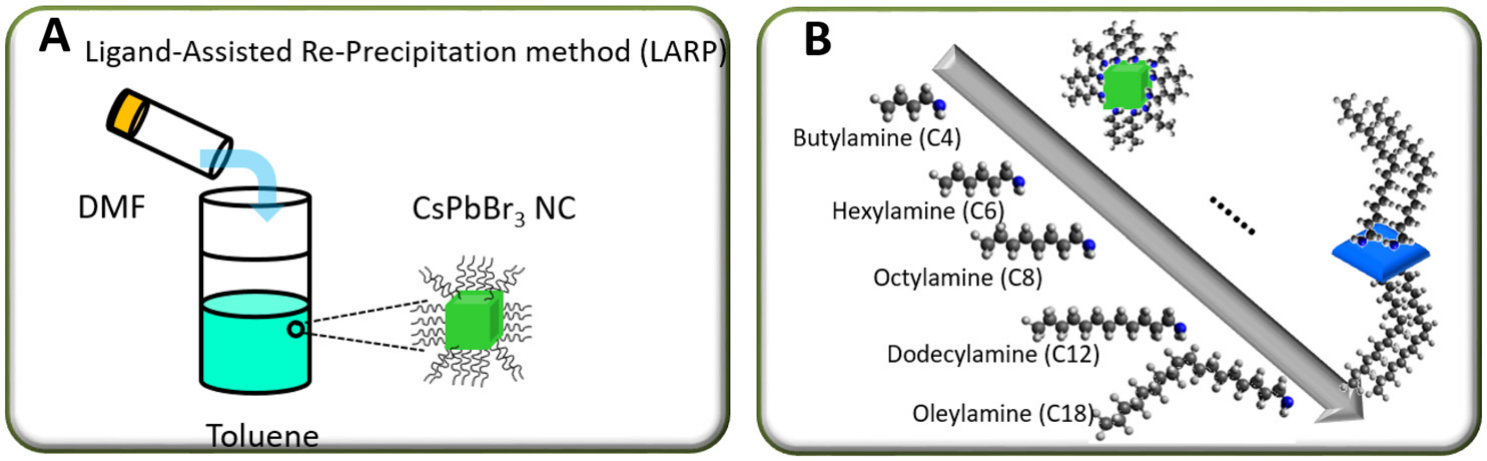
(A) Schematic illustration for synthesis of cesium lead bromide nanocrystals through ligand-assisted reprecipitation method (LARP) wherein the solubility change in the precursor solution leads to the precipitation and recrystallization of corresponding colloidal nanocrystals at room temperatures. (B) Control over the lead octahedral layer thickness of nanocubes vs nanoplatelets through ligand-mediated synthesis using different chain-length of ligands from butylamine (C4) to oleylamine (C18).

**Fig. 2:**
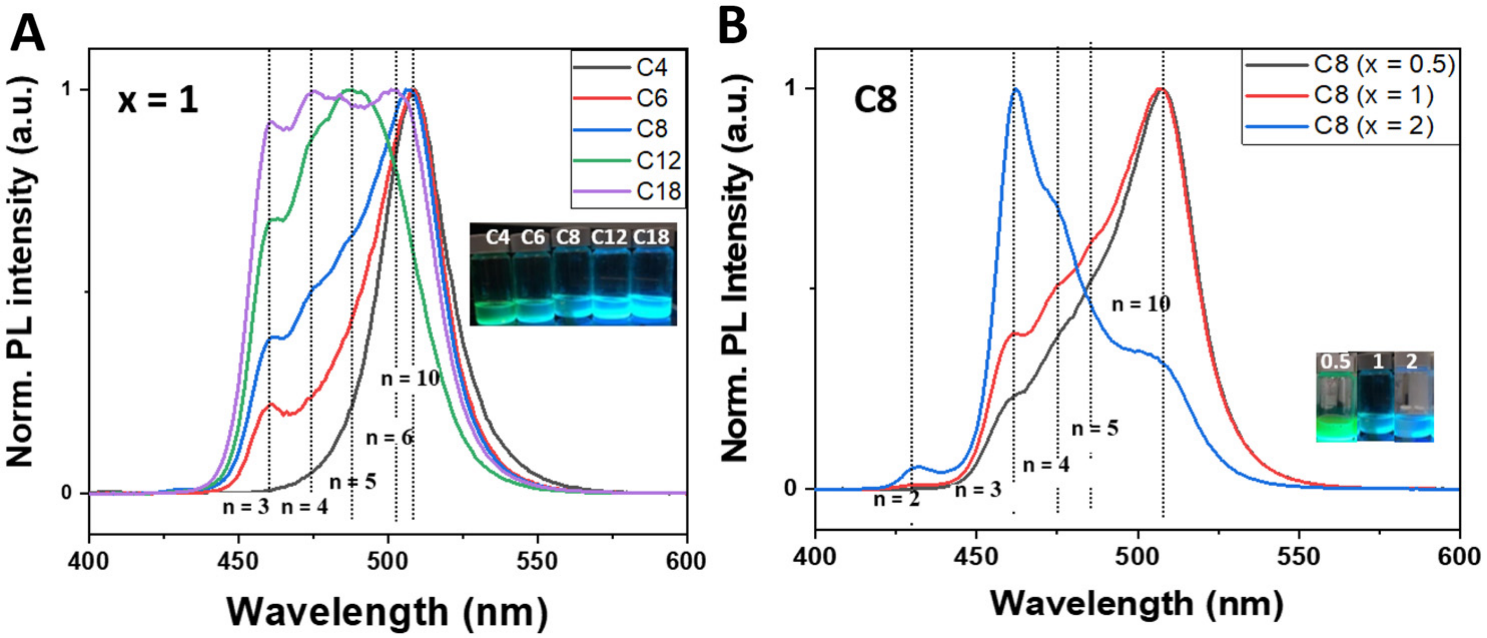
(A-C) Photoluminescent (PL) emission spectra acquired for cesium lead bromide nanocrystals stabilized with different alkylamine chain length from butylamine (C4) to oleylamine (C18) at the Pb:alklaylmine=1:x (*x* = 0.5 (A), *x* = 1 (B), and x= 2 (C)). Inset to individual figure panels represent the digital photograph taken under UV lamp excitation of 365 nm. The dotted lines indicates the different layer thickness of nanoplatelets with n (*n* = 1–10).

**Fig. 3:**
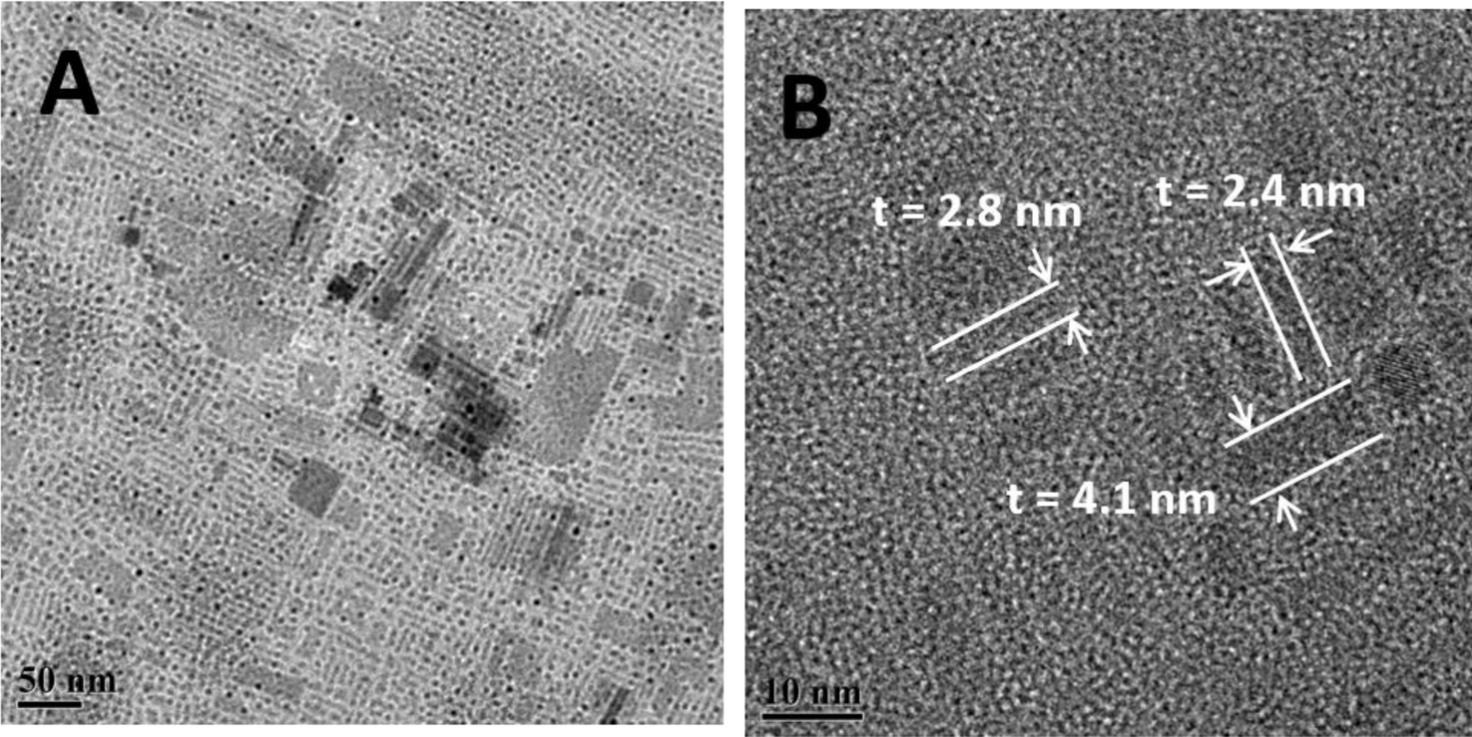
(A) Low-magnification and (B) high-magnification transmission electron microscope (TEM) images acquired for the cesium lead bromide nanoplatelets stabilized using octylamine (C8) as a surface-capping ligands with a concentration of *x* = 2. In panel B, the individual thickness of the nanoplatelets is shown.

**Fig. 4:**
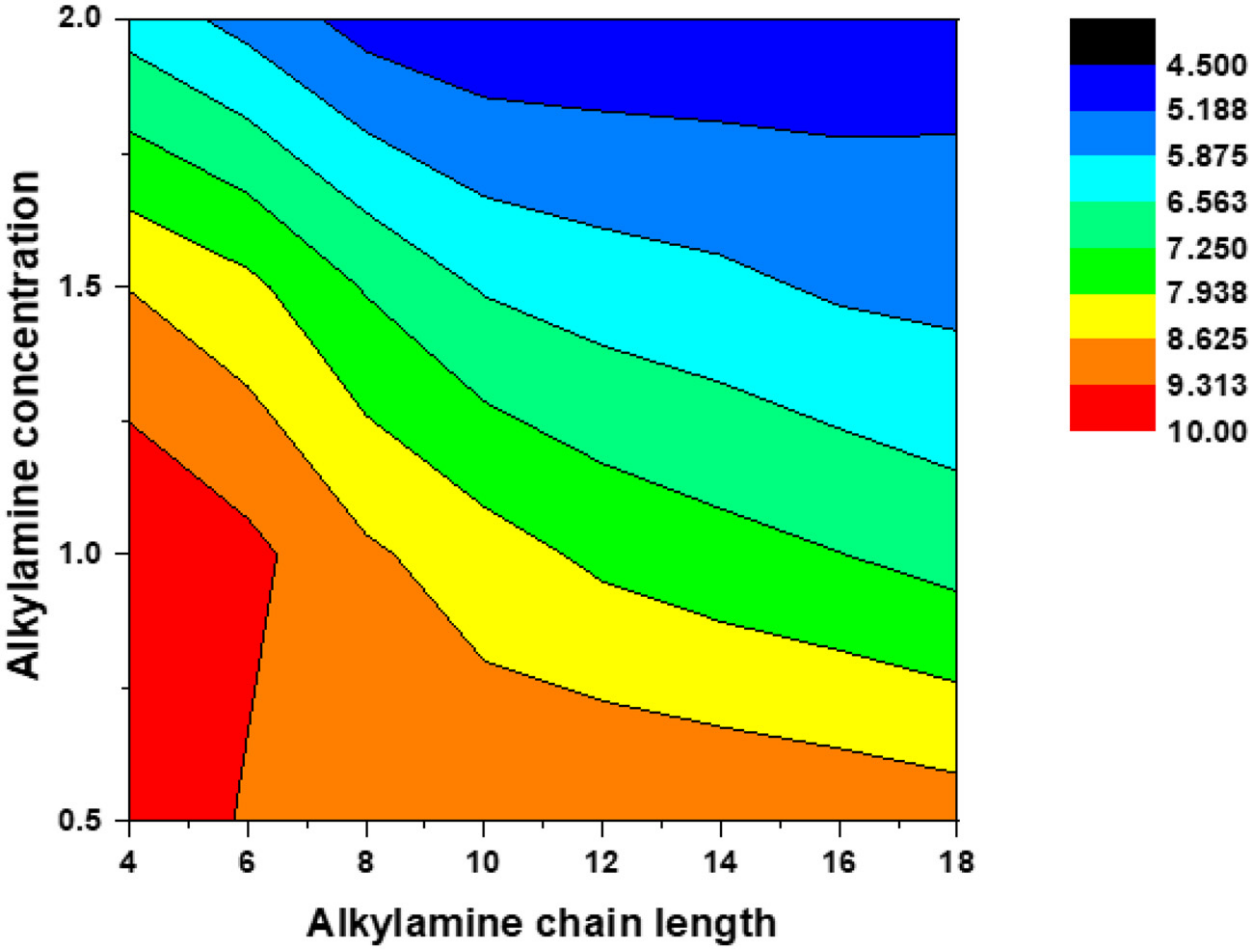
Two-dimensional color contour plots mapping for the average layer thickness of cesium lead bromide nanoplatelets stabilized at varying the alkylamine ligand concentration (*x* = 0.5–2) and chain length (C4-C18). Color bar corresponds to average layer thickness (bluer is thinner while redder is thicker).

**Fig. 5:**
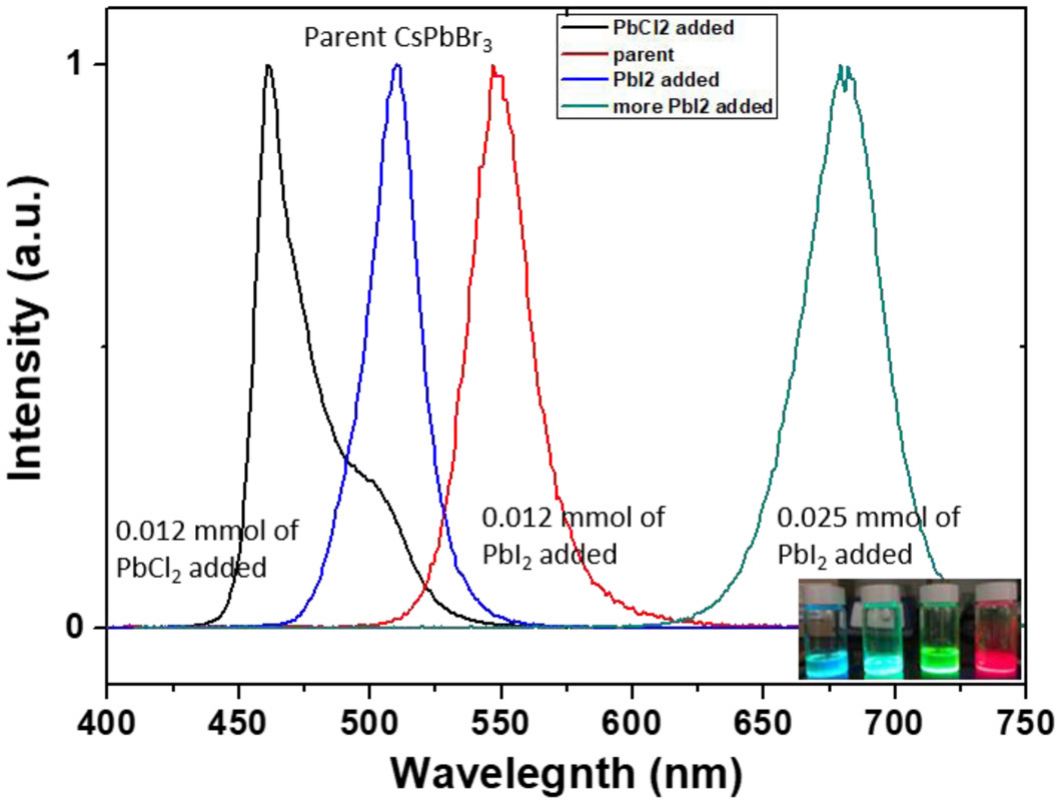
Photoluiminescent emission spectra obtained through halide exchange reaction using thick cesium lead bromide nanoplatelets (C8 with *x* = 0.5) as a parent compound. Inset to figure panel corresponds to the digital photographs for the four different PL spectrum.

The data presented here is related to experimental synthesis of cesium lead bromide nanoplatelets with various thickness of *n* = 2–10 that is accessible through the ligand-assisted reprecipitation (LARP) that is performed at room temperatures. A rapid change in solubility of precursor solution through transferring the solution from a polar solvent to a nonpolar solvent can indeed induce the rapid precipitation and recrystallization of cesium lead bromide. During the process, the alkylamine serving as surface-passivating ligands play a crucial role in stabilizing the different layer thickness of nanoplatelets. A conceptual scheme illustrated in Fig. 1 demonstrates the overall ligand-assisted reprecipitation process to obtain the colloidal cesium lead bromide nanocrystals as a function of varying the alkylamine concentration and chain length. Fig. 2 plots photoluminescne (PL) emission spectra of the acquired cesium lead bromide nanoplatelets that are stabilized at different ligand chain length from butylamine (C4) to oleylamine (c18) with fixed ligand concentration of *x* = 1 and/or at the different ligand concentration of *x* = 0.5 – 2 using C8 as a ligand. The PL emission spectra and emission color under hand UV excitation (365nm) can be used as a proxy to determine the layer thickness (and bandgap) of the nanoplatelets. Fig. 3 shows the transmission electron microscope (TEM) images for the cesium lead bromide nanoplatelets obtained using octylamine (C8) as a ligand at *x* = 2. Fig. 4 plots the 2D color contour mapping for the average layer thickness of cesium lead bromide nanoplatelets obtained at varying the alkylamine ligand concentration (*x* = 0.5–2) and chain length (C4-C18). Fig. 5 illustrates the photoluiminescent emission spectra during the process of halide exchange reactions using the thick cesium lead bromide nanoplatelets (C8 with *x* = 0.5) as a parent compound.

## 2 Chemicals

All chemicals were used without any further purification. Oleic acid (technical grade, 90%), oleylamine (technical grade, 70%), and 1-octadecene (technical grade, 90%) were purchased from Sigma-Aldrich. *n*-octylamine (C8, >98.0%) was purchased from TCI-America. Toluene was purchase from EMD Millipore. Cesium carbonate (Cs_2_CO_3_, 99%), lead bromide (PbBr_2_, 98+%), butylamine (C4, 99%), hexylamine (C6, 99%), dodecylamine (C12, 97%), dimethylformamide (DMF, 99%), and toluene (anhydrous, 99.8%) were purchased from Alfa-Aesar.

## 3 Experimental Design, Materials and Methods

The synthesis and data acquisition for colloidal cesium lead bromide nanoplatelets have been demonstrated in the previously reported publications [1], [2], [3]. Coresponding colloidal nanocrystals were stabilized through the ligand-assisted reprecipitation method (LARP) performed at room temperature [4]. Briefly, a precursor solution including Cs-oleate and lead bromide (PbBr_2_) was made by mixing 37.5 uL of 0.4 M Cs-oleate, 0.030 mmol (0.011 g) of PbBr_2_, and 0.2 mL of oleic acid which are consequently dissolved in 0.6 mL DMF with a stoichiometric amount addition of alkylamine ligands (C4-C18); 0.4M of Cs-oleate solution was already made by mixing and dissolving 2.5 mmol (0.4072 g) of Cs_2_CO_3_ in 2.5 mL of oleic acid and 10 mL ODE that were further heated at 180 °C under ambient condition. The stoichiometric ratio of Pb:alkylamine was systemetically varied between *x* = 0.5 and 2. As-prepared mixed precursor solution was rapidly transferred to 10 mL of tolune with vigorous stirring which also leads to the formation of cesium lead bromide nanoplatelets. The crude solution was centrifuged at 5000 rpm for 10 min to obtain the precipitation, which was then re-dispersed in 5 mL of toluene for furthre characterziations. Halide exchange reactions were then performed using the resulting CsPbBr3 nanocrystal dispersion in toluene as a parent solution by addiing a stoichiometic amount of lead halide sources (such as PbCl_2_ and PbI_2_ that are already prepard with mixing with oleic acid, oleylamine, and octadecene and heating at 200 °C until entire dissolution of solids). Photoluminescence (PL) emission spectra were recorded with a Horiba PTI Quanta-Master series spectrofluorometer with a Xenon arc lamp as the light excitation source and a photomultiplier tube detector. The excitation wavelength of 360 nm was chosen to record the corresponding PL emission spectra. High- and low-magnification transmission electron microscopy (TEM) images were collected using a FEI Tecnai G2 F20 ST instrument opertaing at an accelated voltage of 200 kV.

## Ethics Statement

The study does not contain any research involving human participants and/or animals performed by any of the author.

## CRediT Author Statement

**Junsang Cho:** Conceptualization, Methodology, Experiment, Data analysis, Writing-Original Draft Preparation, Investigation, Supervision, Software, Writing- Reviewing and Editing

## Declaration of Competing Interest

The authors declare that they have no known competing financial interests or personal relationships which have or could be perceived to have influenced the work reported in this article.

## Data Availability

Dataset of Ligand-Controlled Synthesis of CsPbBr3 Nanoplatelets (Original data) (Mendeley Data).
